# The GLP1R Agonist Semaglutide Inhibits Reactive Astrocytes and Enhances the Efficacy of Neural Stem Cell Transplantation Therapy in Parkinson's Disease Mice

**DOI:** 10.1002/advs.202417664

**Published:** 2025-08-28

**Authors:** Dan Song, Xiaoya Zou, Di Ma, Yuying Zhao, Tingting Liu, Bibiao Shen, Oumei Cheng

**Affiliations:** ^1^ Department of Neurology The First Affiliated Hospital of Chongqing Medical University Chongqing 400016 China; ^2^ Department of Rehabilitation The Second Affiliated Hospital of Chongqing Medical University Chongqing 400010 China; ^3^ Laboratory Research Center The First Affiliated Hospital of Chongqing Medical University Chongqing 400016 China; ^4^ Key Laboratory of Major Brain Disease and Aging Research (Ministry of Education) Chongqing 400016 China

**Keywords:** astrocytes, cell transplantation, GLP1R agonist, neural stem cells, parkinson's disease

## Abstract

Cell transplantation offers a promising approach for treating Parkinson's disease (PD), but the limited survival of transplanted cells remains a major challenge. Reactive astrocytes, abundant in PD brains, may exacerbate this issue. GLP1R agonists, like semaglutide, are shown to inhibit reactive astrocytes in PD models. This study explores whether semaglutide could enhance the survival of transplanted neural stem cells (NSCs) in PD treatment. Six‐hydroxydopamine‐induced PD mouse models are used, with midbrain‐derived NSCs transplanted into the lesioned striatum. Semaglutide is administered every other day for four weeks. In vivo imaging tracks the survival and distribution of DiD‐labeled NSCs, while differentiation and astrocyte phenotypic changes are examined. Results show that semaglutide combined with NSC transplantation improves motor function. The mean fluorescence photon flux of mice transplanted with DiD‐labeled NSCs alone is 0.8192 × 10^−11^, compared to 3.258 × 10^−11^ in those receiving both semaglutide and NSCs. Additionally, semaglutide reduces C3^+^ reactive astrocytes (previously A1 reactive astrocytes) in the striatum. Co‐culture experiments indicate that C3^+^ reactive astrocytes hinder NSCs differentiation. RNA‐seq reveals enriched inflammatory factors in C3^+^ astrocytes. Semaglutide combined with NSCs transplantation may enhance PD treatment partly by inhibiting C3^+^ reactive astrocytes and promoting the survival and differentiation of transplanted cells.

## Graphical abstract

Compared to transplantation of neural stem cells (NSCs) alone, the combined treatment of NSCs with semaglutide more effectively improves motor dysfunction in Parkinson's disease (PD) mice. This synergistic effect may result from semaglutide's ability to suppress microglial activation, thereby preventing the transformation of resting astrocytes into harmful C3^+^ reactive astrocytes, ultimately enhancing the survival and differentiation of transplanted NSCs.

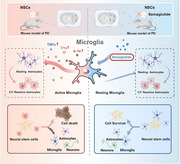

## 1 Introduction

Parkinson's disease (PD) is a neurodegenerative disorder associated with aging, characterized by the death of dopamine (DA) neurons in the midbrain and a reduction in DA levels in the striatum.^[^ ^1^, ^2^ ^]^ Clinically, PD presents with motor symptoms such as tremors, rigidity, bradykinesia, and postural instability, along with a range of non‐motor symptoms.^[^ ^3^, ^4^, ^5^ ^]^ Currently, there is no conclusive evidence supporting the efficacy of existing therapies in slowing the progression of the disease.^[^ ^1^, ^3^, ^5^ ^]^ Hence, there is an urgent need to explore effective treatment strategies for PD.

The development of cell transplantation technology offers promise for the treatment of PD and is a current research hotspot.^[^ ^6^, ^7^, ^8^ ^]^ Neural stem cells (NSCs) display remarkable proliferative and differentiative potential, ease of integration within the host, reduced susceptibility to immune rejection, adaptability for gene modification, and a relatively low risk of tumorigenesis, making them an ideal candidate for cell therapies. Theoretically, NSCs transplantation may restore their original physiological functions and achieve therapeutic outcomes by secreting trophic factors, immune regulation, and differentiating into specific cell types to replace lost neurons. However, NSCs transplantation faces significant challenges, particularly regarding low cell survival.^[^ ^9^, ^10^, ^11^ ^]^ Thus, a critical focus of current research is how to enhance the survival rate of transplanted cells, enabling them to establish normal synaptic connections with surrounding cells and effectively integrate into neural circuits.

The survival, differentiation, and maturation of NSCs are largely regulated by non‐cell autonomous mechanisms within their microenvironment.^[^ ^12^, ^13^ ^]^ Astrocytes, the most abundant and widely distributed glial cells in the mammalian brain, play a pivotal role in regulating central nervous system (CNS) homeostasis. Resting astrocytes serve as nutritional support cells in the CNS, but when the CNS is damaged, they become activated and transform into reactive astrocytes, undergoing changes in their morphology, number, and biological functions.^[^ ^14^ ^]^ Growing evidence suggests that astrocytes exhibit morphological and functional heterogeneity, particularly the recently identified disease‐associated C3^+^ astrocytes,^[^ ^15^, ^16^, ^17^, ^18^, ^19^ ^]^ previously simplified as neurotoxic (A1).^[^ ^20^, ^21^ ^]^ Functionally, C3^+^ astrocytes lose many normal astrocytic functions and may induce apoptosis in various neuronal types through the release of specific neurotoxic factors.^[^ ^10^, ^22^ ^]^ Moreover, a significant accumulation of pro‐inflammatory C3^+^ astrocytes has been observed in the brain tissue of patients with PD.^[^ ^20^, ^23^ ^]^ Recent studies have shown that co‐transplantation of autologous regulatory T cells can suppress acute neuroinflammation and immune cell infiltration, protect transplanted cells, and improve the prognosis of PD models.^[^ ^9^ ^]^ This further indicates a close relationship between the survival of transplanted cells and the inflammatory microenvironment. Based on this, we hypothesize that C3^+^ reactive astrocytes, as an important component of the microenvironment, may influence the survival and integration of transplanted NSCs through non‐cell‐autonomous toxic effects.

Glucagon‐like peptide‐1 receptor (GLP1R) agonists have anti‐inflammatory, neurotrophic, and neuroprotective properties in preclinical models of neurodegenerative diseases and are considered promising neuroprotective agents in neurological disorders.^[^ ^24^, ^25^, ^26^ ^]^ Studies have found that GLP1R agonists may exert neuroprotective effects by reducing the transformation of astrocytes into neurotoxic phenotypes.^[^ ^27^ ^]^ Semaglutide (SEG) is a GLP1R agonist.^[^ ^28^ ^]^ This study aims to enhance the effectiveness of NSCs transplantation therapy for PD by inhibiting C3^+^ astrocytes through the combination of SEG and NSCs, offering a novel approach to PD treatment. Our findings demonstrated that the combined transplantation of SEG and NSCs effectively prevents the conversion of astrocytes to the C3^+^ phenotype and ameliorates motor impairments in 6‐Hydroxydopamine (6‐OHDA)‐induced PD mice. In conclusion, our research provides a new perspective on the treatment of PD.

## 2 Results

### 2.1 Astrocyte Phenotypic Transformation and Impaired Endogenous Neurogenesis in PD Mice

**Figure 1:**
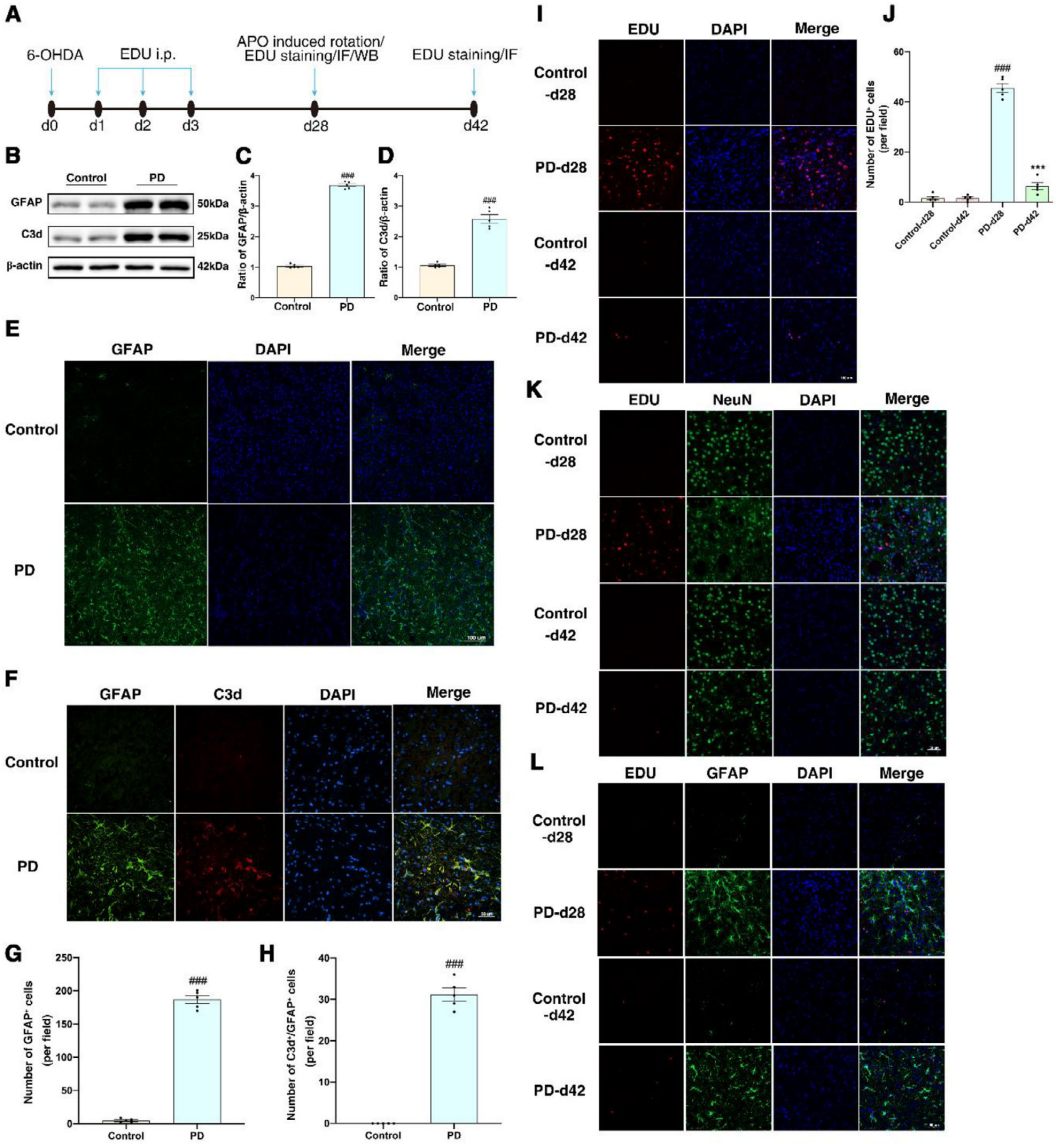
The transformation of astrocyte phenotype to C3^+^ phenotype and endogenous neurogenesis were impaired in PD mice. A) The timelines of the animal experiment 1. B) Representative bands of GFAP and C3d expression in the striatum detected by Western Blot. C,D) Statistical graphs of GFAP and C3d expression in the striatum detected by Western Blot. E) Representative immunofluorescence image of GFAP^+^ cells expression in the striatum. Scale bar: 100 µm. F) Representative immunofluorescence image of GFAP^+^/C3d^+^ cell expression in the striatum. Scale bar: 50 µm. G) Quantitative analysis statistical graph indicates a significant increase in the number of GFAP^+^ cells in the PD group. H) Quantitative analysis statistical graph indicates a significant increase in the number of GFAP^+^/C3d^+^ cells in the PD group. I) Differential time‐point representative immunofluorescence images of EDU^+^ cell expression in the striatum. Scale bar: 100 µm. J) Quantitative analysis statistical charts indicate that, compared to the Control group (d28), the number of EDU^+^ cells in the striatum of PD group (d28) mice significantly increased. However, in the PD group mice 42 days post‐surgery, there was a significant decrease in the number of EDU^+^ cells compared to 28 days post‐surgery. K) Representative immunofluorescence images show non‐co‐localization of EDU^+^ cells with NeuN^+^ cells in the striatum. Scale bar: 50 µm. L) Representative immunofluorescence images show that EDU^+^ cells in the striatum hardly colocalize with GFAP^+^ cells. Scale bar: 50 µm. ^###^ *p*<0.001 compared with the Control group. ^^^*p*<0.001 compared with the Control‐d28 group. ^***^ *p*<0.001 compared with the PD‐d28 group. (*n* = 5 samples per condition; unpaired Student's *t*‐test with Kolmogorov‐Smirnov test was used to compare the two groups, and one‐way ANOVA with Tukey post‐test was used for the rest).

The timeline of Animal Experiment 1 is shown in **Figure** **1**A. To validate the successful induction of the PD model in mice by stereotactic injection of 6‐OHDA into the striatum, we performed a series of experiments. The APO‐induced rotation test showed that, compared to the Control group, mice in the PD group exhibited rotational behavior toward the non‐lesioned side, using either the forelimbs or hindlimbs as the supporting points, with the body forming a circular posture and a rotation speed >7 r min^−1^ (Figure S1A, Supporting Information). The protein expression levels of TH in the PD group were significantly lower compared to the Control group (Figure S1B,C, Supporting Information). Immunofluorescence staining results showed that the number of TH^+^ cells in the substantia nigra and the fluorescence intensity of TH in the striatum were significantly reduced in the PD group compared to the Control group (Figure S1D–F, Supporting Information).

To determine whether the 6‐OHDA‐induced PD mouse model induces changes in astrocyte phenotypes and neurogenesis, we characterized astrocytic phenotypic alterations and quantified newly generated cells in the striatum at different time points. Compared to the control group, the protein expression levels of GFAP and C3d were significantly increased in the PD group. (Figure 1B–D). Immunofluorescence staining results showed a significant increase in GFAP^+^ cells and C3d^+^/GFAP^+^ cells in the striatum of mice in the PD group compared to the Control group (Figure 1E–H). Four weeks after stereotactic injection of 6‐OHDA into the brain, the number of EDU^+^ cells in the striatum of the PD group mice was significantly increased compared to the Control group, while EDU^+^ cells almost did not co‐localize with NeuN^+^ cells or GFAP^+^ cells (Figure 1I–L). Compared with the PD‐d28 group (4 weeks after intracerebral stereotactic injection of 6‐OHDA), the PD‐d42 group (6 weeks post‐injection) showed a significant reduction in the number of EDU^+^ cells in the striatum, with minimal co‐localization observed between EDU^+^ cells and NeuN^+^ or GFAP^+^ cells (Figure 1I–L).

### 2.2 GLP1R Agonist Combined with NSCs Transplantation Therapy Improved the Motor Behavior of PD Mice

**Figure 2:**
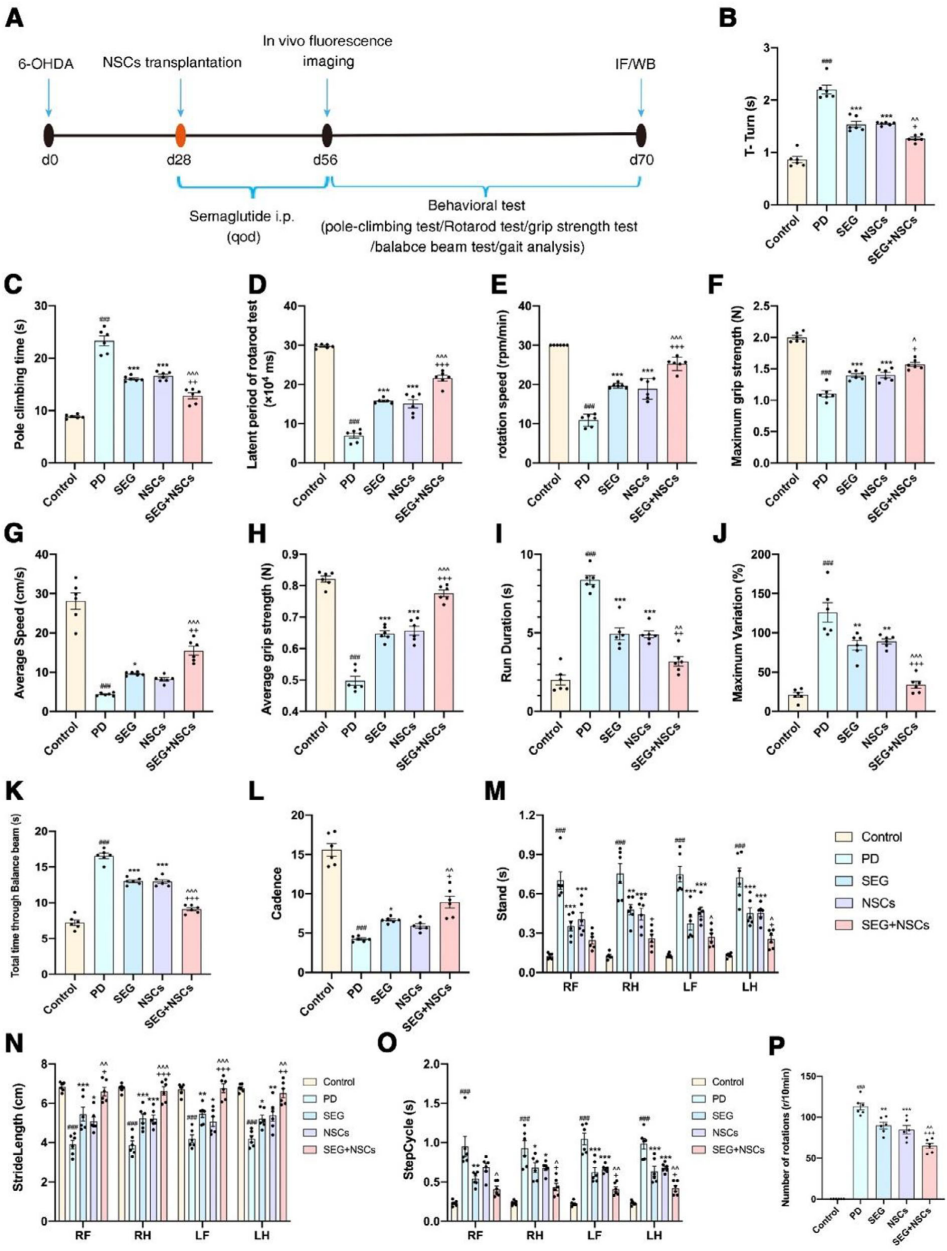
GLP1R agonist combined with NSCs transplantation therapy improved the motor behavior of PD mice. A) The timelines of the animal experiment 2. B) Pole climbing test T‐Turn time in pole climbing test. C) Total pole climbing time in the pole climbing test. D) Latency period in the Rotarod test. E) Drop Speed in Rotarod test. F) Maximum grip strength in the grip strength test. G) Average grip strength in the grip strength test. H) Crossing the balance beam in the balance beam test. I) The Duration of the gait test. J) The maximum variation in the gait test. K) The average speed in the gait test. L) The cadence in gait test. M) The support phase time (stand) of the limbs in the gait test. N) The stridelength of the limbs in the gait test. O) The step cycle of the limbs in the gait test. P) The number of rotations induced by APO. ^###^ *p*<0.001 compared with the Control group; ^*^ *p*<0.05 compared with the PD group; ^**^ *p*<0.01 compared with the PD group; ^***^ *p*<0.001 compared with the PD group; ^*p*<0.05 compared with the NSCs group; ^^*p*<0.01 compared with the NSCs group; ^^^*p*<0.001 compared with the NSCs group; ^+^ *p*<0.05 compared with the SEG group; ^++^ *p*<0.01 compared with the SEG group; ^+++^ *p*<0.001 compared with the SEG group. RF: right front; RH: right hind; LF: left front; LH: left hind. (*n* = 6 samples per condition; one‐way ANOVA with Tukey post‐test).

The timeline of the animal experiment 2 is shown in **Figure** **2**A. To assess the potential of combined SEG and NSC transplantation in improving motor function in PD mice, we conducted a series of behavioral experiments four weeks post‐transplantation to measure their spontaneous activity and motor coordination. The results of the pole‐climbing experiment indicated that mice in the PD group had significantly prolonged head‐turning time (T‐Turn) and pole climbing time compared to the Control group (Figure 2B,C). In contrast, mice in the SEG group and NSCs group showed reduced T‐Turn and pole climbing time relative to the PD group (Figure 2B,C). Moreover, compared to the NSCs group, mice in the SEG+NSCs group had further reduced T‐Turn and pole climbing time (Figure 2B,C).

The results of the Rotarod test showed that, compared to the Control group, both the time spent on the rod and the speed at which the mice fell off were decreased in the PD group (Figure 2D,E). In contrast, the SEG and NSCs groups exhibited increased time on the rod and higher speeds before falling off compared to the PD group (Figure 2D,E). Furthermore, the SEG+NSCs group showed an increase in both the time on the rod and the speed at which the mice fell off when compared to the NSCs group (Figure 2D,E).

The results of the grip strength test indicated that, compared to the Control group, both the maximum and average grip strength were significantly reduced in the PD group (Figure 2F,G). Conversely, mice in the SEG and NSCs groups showed increased maximum and average grip strength relative to the PD group (Figure 2F,G). Notably, mice in the SEG+NSCs group exhibited further improvements in both measures compared to the NSCs group (Figure 2F,G).

The results of the balance beam test showed that mice in the PD group took significantly more time to cross the beam compared to the Control group (Figure 2H). Mice in the SEG and NSCs groups required less time to traverse the balance beam than those in the PD group. (Figure 2H). Moreover, mice in the SEG+NSCs group exhibited further reductions in crossing time compared to the NSCs group (Figure 2H).

The results of the gait test indicated that, compared to the Control group, both the time to cover a specific distance (Duration) and the maximum variation rate (Maximum Variation) were significantly increased in the PD group (Figure 2I,J). Compared to the PD group, both Duration and Maximum Variation were decreased in the SEG and NSCs groups (Figure 2I,J). Furthermore, the SEG+NSCs group exhibited further reductions in both Duration and Maximum Variation compared to the NSCs group (Figure 2I,J). Compared to the Control group, the Average Speed and number of steps per unit time (Cadence) were significantly reduced in the PD group (Figure 2K,L). Compared to the PD group, both Average Speed and Cadence were increased in the SEG and NSCs groups (Figure 2K,L). Moreover, Average Speed and Cadence in the SEG+NSCs group were further increased compared to the NSCs group (Figure 2K,L).

Compared to the Control group, the support phase duration (Stand) of the limbs was significantly prolonged in the PD group. In contrast, the stance of the limbs was reduced in the SEG and NSCs groups relative to the PD group. The Stand of the left forelimb and left hindlimb was further decreased in the SEG+NSCs group compared to the NSCs group (Figure 2M). Compared to the Control group, the stride length of the limbs was significantly reduced in the PD group. The StrideLength of the limbs increased in the SEG and NSCs groups relative to the PD group, with a further significant increase observed in the SEG+NSCs group compared to the NSCs group (Figure 2N). Additionally, the StepCycle of the limbs was significantly prolonged in the PD group compared to the Control group. However, the StepCycle of the limbs was reduced in the SEG group relative to the PD group, with decreases observed in the left forelimb, left hindlimb, and right hindlimb in the NSCs group. Compared to the NSCs group, the StepCycle of the limbs in the SEG+NSCs group exhibited further decreased (Figure 2O).

The APO‐induced rotation test revealed a marked increase in rotational counts in the PD group relative to the control group (Figure 2P). In contrast, both SEG and NSCs monotherapies significantly attenuated APO‐evoked rotations compared with the PD group (Figure 2P). Furthermore, combined SEG+NSCs treatment elicited an additional reduction in rotational behavior beyond that observed in the NSCs group (Figure 2P).

Our findings demonstrate that the combination of the GLP1R agonist semaglutide and NSC transplantation treatment can further improve motor function in PD mice, compared to NSC transplantation alone.

### 2.3 GLP1R Agonist Treatment Improved Survival and Differentiation of Transplanted NSCs

**Figure 3:**
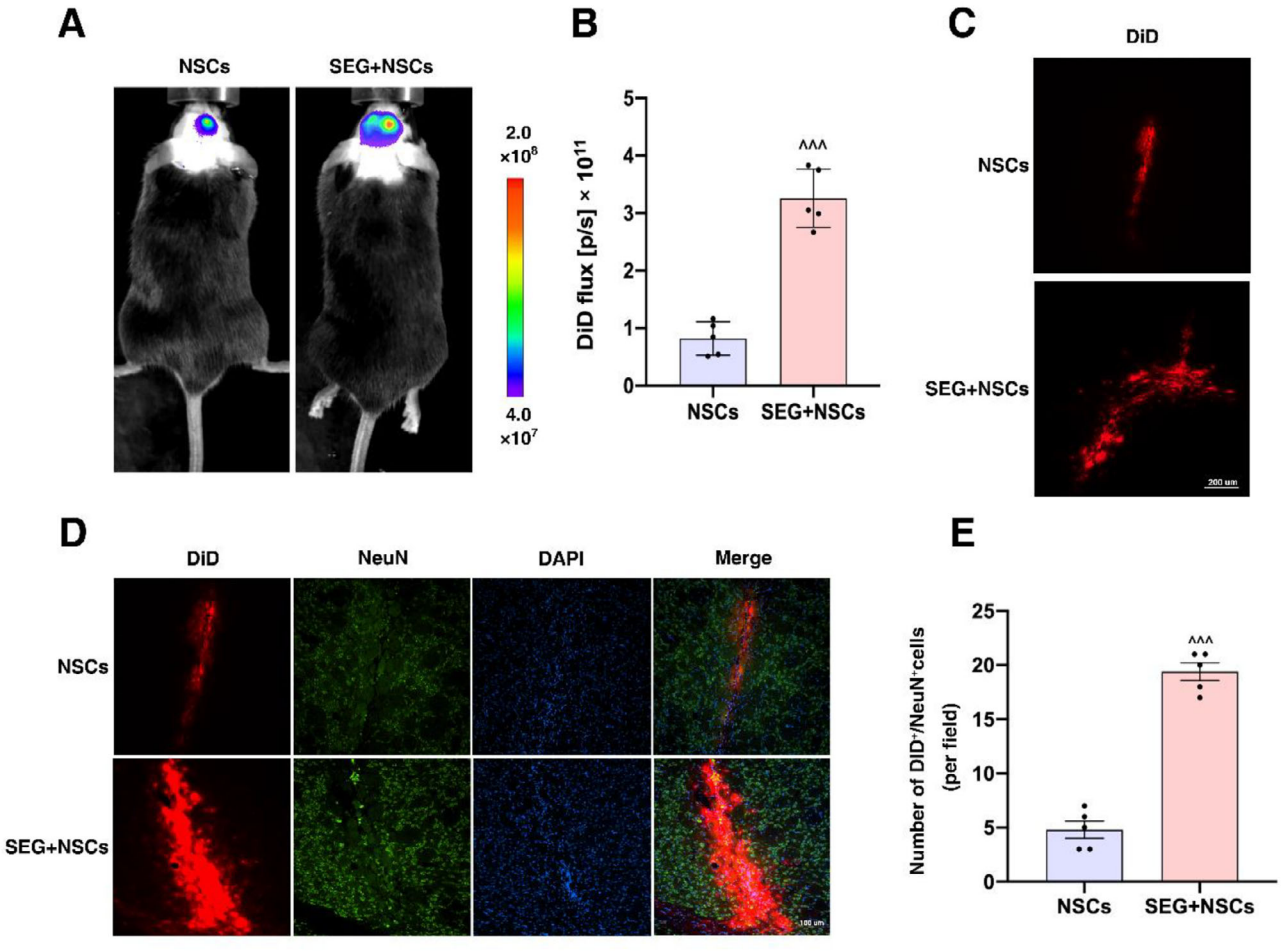
GLP1R agonist treatment improved the survival and differentiation of transplanted NSCs. A) Representative images of in vivo fluorescence imaging techniques. B) Quantitative analysis of photon flux in fluorescence imaging in vivo. C) DiD+ cell clusters in each group. Scale bar: 200 µm. D) Representative images of DiD^+^ cells and NeuN^+^ cells. Scale bar: 100 µm. E) Statistical graph of DiD^+^/NeuN^+^ cells in striatum. ^^^*p*<0.001 compared with the NSCs group. (*n* = 5 samples per condition; unpaired Student's *t*‐test with Kolmogorov‐Smirnov test).

Using in vivo imaging technology to trace DiD fluorescent dye‐labeled NSCs, we determined their survival and distribution in the brain. In the NSCs group, transplanted cells colonized around the transplantation target, whereas in the SEG+NSCs group, the transplanted cells not only concentrated at the target site but also extensively migrated to surrounding areas on both the ipsilateral and contralateral sides (**Figure** **3**A). Quantitative analysis of the in vivo imaging results showed that, compared to the NSCs group, the photon flux of the fluorescent imaging significantly increased in the SEG+NSCs group (Figure 3B). Brain slice imaging revealed that, compared to the NSCs group, the SEG+NSCs group had a significant increase in DiD^+^ cell clusters (Figure 3C) and an increased number of DiD and NeuN co‐labeled cells (Figure 3D,E). These results indicated that SEG treatment facilitates the survival and differentiation of transplanted cells.

### 2.4 GLP1R Agonists Combined with NSCs Transplantation Reduced Inflammatory Astrocyte Expression in PD Mice

**Figure 4:**
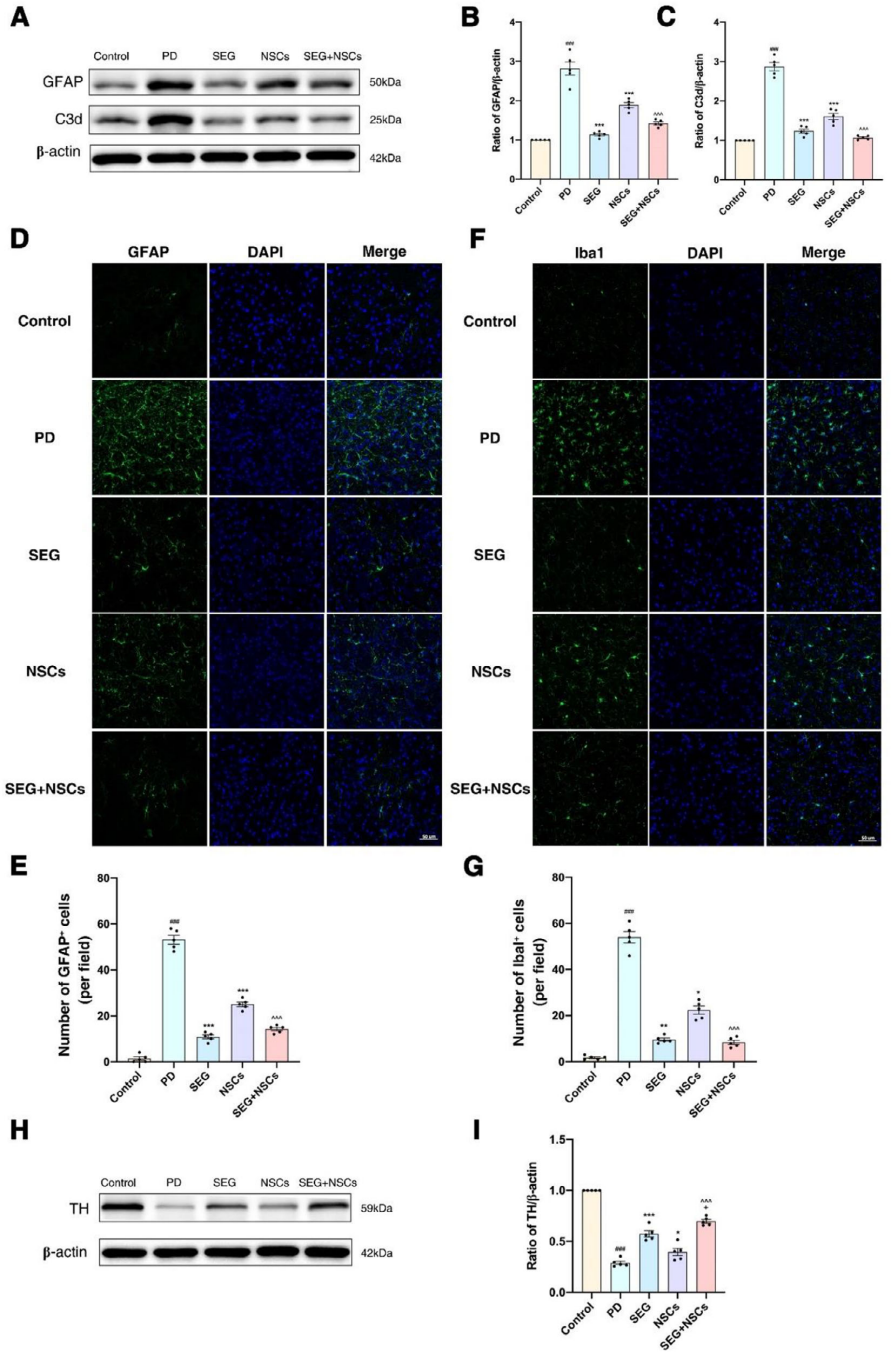
GLP1R agonists combined with NSCs transplantation reduced the expression of inflammatory astrocytes in PD mice. A) The representative bands of GFAP/C3d expression in the striatum of each group were detected by Western blot. B,C) Statistical graph of GFAP/C3d expression in striatum detected by Western Blot. D) Representative immunofluorescence image of GFAP+ cells expression in the striatum. Scale bar: 50µm. E) Statistical graph of GFAP+ cells in the striatum. F) Representative immunofluorescence image of Iba1^+^ cells expression in the striatum. Scale bar: 50 µm. G) Statistical graph of Iba1^+^ cells in striatum. H) The representative bands of TH expression in the striatum of each group were detected by Western blot. I) Statistical graph of TH expression in the striatum detected by Western Blot. ^###^ *p*<0.001 compared with the Control group; ^*^ *p*<0.05 compared with the PD group; ^**^ *p*<0.01 compared with the PD group; ^***^ *p*<0.001 compared with the PD group; ^^^*p*<0.001 compared with the NSCs group; ^+^ *p*<0.05 compared with the SEG group. (*n* = 5 samples per condition; one‐way ANOVA with Tukey post‐test).

Our study found that, compared to the Control group, protein expression levels of C3d and GFAP were significantly elevated in the PD group, along with a marked increase in the number of GFAP^+^ cells (**Figure** **4**A–E). Compared to the PD group, both the protein expression levels of C3d and GFAP and the number of GFAP^+^ cells were significantly reduced in the SEG and NSCs groups (Figure 4A–E). Furthermore, in the SEG+NSCs group, protein expression levels of C3d and GFAP were further diminished, and the number of GFAP^+^ cells was further reduced compared to the NSCs group (Figure 4A–E).

Simultaneously, microglial activation in each group was assessed via immunofluorescence. This study found that, compared to the Control group, the number of Iba1^+^ cells was significantly increased in the PD group (Figure 4F,G). The number of Iba1+ cells was reduced in both the SEG and NSCs groups relative to the PD group (Figure 4F,G). Furthermore, compared to the NSCs group, the number of Iba1^+^ cells in the SEG+NSCs group mice further decreased (Figure 4F,G). Immunoblotting to detect pathology showed that, compared to the Control group, the protein expression of TH was significantly decreased in the PD group (Figure 4H,I). TH protein expression was increased in both the SEG and NSCs groups compared to the PD group (Figure 4H,I). Moreover, the SEG+NSCs group exhibited a further increase in TH protein expression compared to the NSCs group (Figure 4H,I).

In addition to glial marker quantification, we performed detailed morphological analyses of astrocytes and microglia. Astrocytes in the PD group exhibited typical features of reactive gliosis, including enlarged cell bodies, shorter and fewer processes, and decreased branching complexity when compared with the Control group (Figure S2A–D, Supporting Information). These morphological abnormalities were partially alleviated in both the SEG and NSCs groups (Figure S2A–D, Supporting Information). Notably, the SEG+NSCs group showed more substantial improvement (Figure S2A–D, Supporting Information).

Similarly, microglia in the PD group predominantly displayed an amoeboid morphology characterized by enlarged soma size, retracted and fewer processes, and reduced total branch number—hallmarks of a pro‐inflammatory state (Figure S2E–H, Supporting Information). These pathological features were ameliorated to varying degrees in both the SEG and NSCs groups (Figure S2E–H, Supporting Information), with the SEG+NSCs group exhibiting the more robust restoration of microglial morphology (Figure S2E–H, Supporting Information).

### 2.5 The Impact of C3^+^ Astrocytes on NSCs

**Figure 5:**
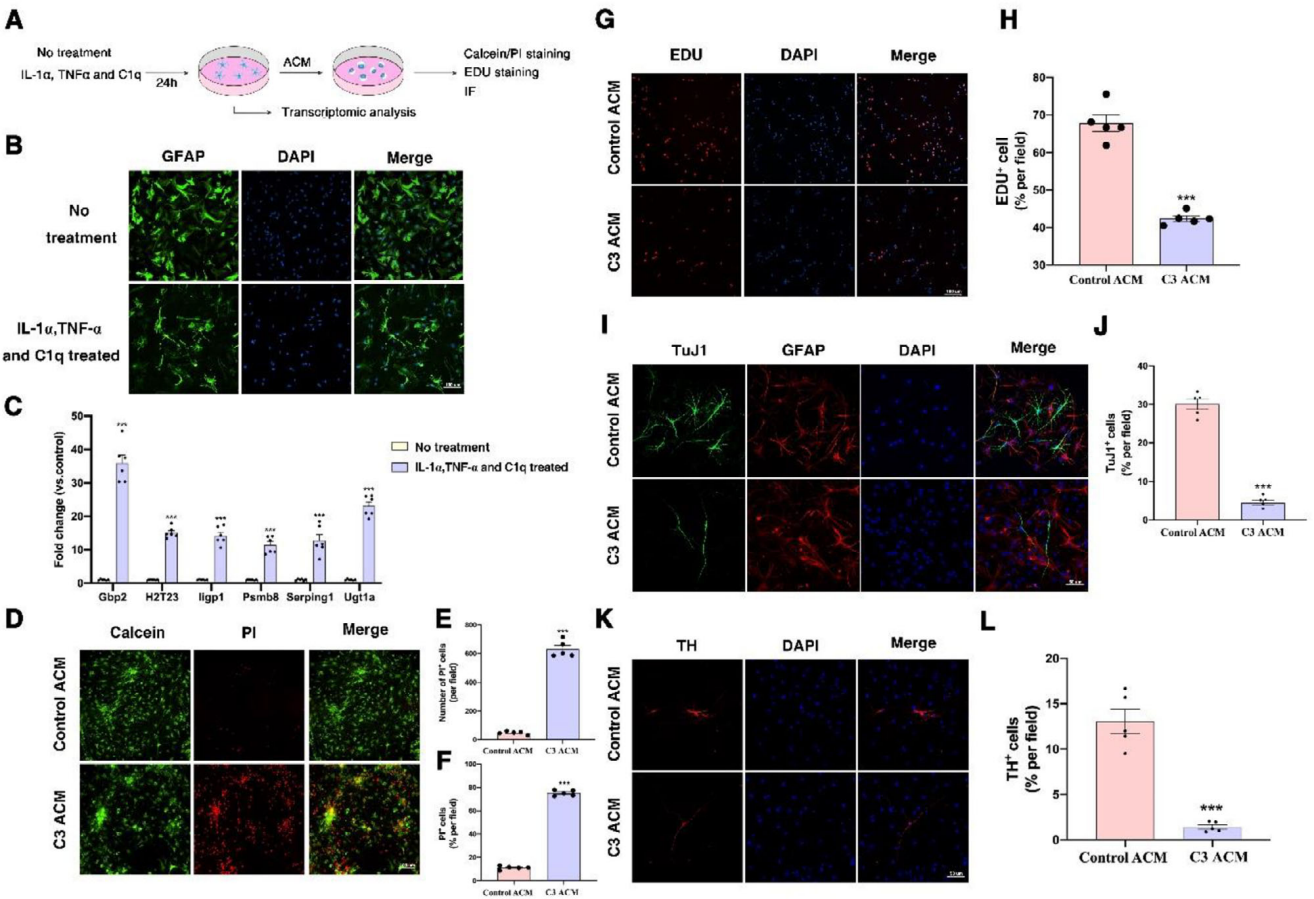
The Impact of C3^+^ astrocytes on NSCs. A) The flowchart for Cell Experiment 1. B) Morphology of different subtypes of astrocytes. Scale bar: 100 µm. C) IL‐1α, TNF‐α, and C1q treatment increased the expression of C3‐related genes in astrocytes. D) Representative fluorescence image of NSCs cell activity and toxicity. Scale bar: 100 µm. E) Statistical graph of dead cell count. F) Statistical graph of dead cell rate. G) Representative fluorescence image of EDU staining of NSCs. Scale bar: 100 µm. H) Statistical graph of EDU^+^ cell rate. I) Representative immunofluorescence image of NSCs differentiation into neurons. Scale bar: 50 µm. J) Statistical graph of Tuj1^+^ cell rate. K) Representative immunofluorescence image of NSCs differentiation into TH^+^ neurons. Scale bar: 50 µm. L) Statistical graph of TH^+^ cell rate. ^***^ *p*<0.001. (*n* = 6/5 samples per condition; unpaired Student's *t*‐test with Kolmogorov‐Smirnov test).

The flowchart for Cell Experiment 1 is shown in **Figure** **5**A. Immunofluorescence staining was used to identify primary cultured astrocytes and NSCs (Figure S3A,B, Supporting Information). Immunofluorescence staining and RT‐qPCR results showed that treatment with IL‐1α, TNFα, and C1q altered the morphology of astrocytes (Figure 5B) and upregulated the expression of genes associated with the C3^+^ phenotype, including Serping1, Gbp2, Iigp1, H2‐T23, Ugt1a, and Psmb8 (Figure 5C). These results indicate successful in vitro induction of C3^+^ astrocytes.

To determine the effects of C3^+^ astrocytes on the survival, proliferation, and differentiation of NSCs, astrocyte‐conditioned medium (ACM) was collected and used for the conditional co‐culture of NSCs. Cell viability and cytotoxicity assays revealed that, compared to the Control ACM group, the number and proportion of PI^+^ cells in the C3‐ACM group NSCs were significantly increased (Figure 5D–F), indicating that C3^+^ astrocytes caused extensive cell death in neural stem cells. The proportion of EDU^+^ cells in the C3‐ACM group NSCs was significantly lower than in the Control ACM group (Figure 5G,H), suggesting that C3^+^ astrocytes hindered the proliferation of NSCs. Additionally, the ratio of TuJ1^+^ cells and TH^+^ cells in the C3‐ACM group NSCs was significantly decreased (Figure 5I–L), indicating a reduction in the differentiation of NSCs into neurons and dopaminergic neurons in the C3‐ACM group.

### 2.6 The Upregulation of Inflammatory Immune and Chemotactic Signaling Pathways in C3^+^ Astrocytes

**Figure 6:**
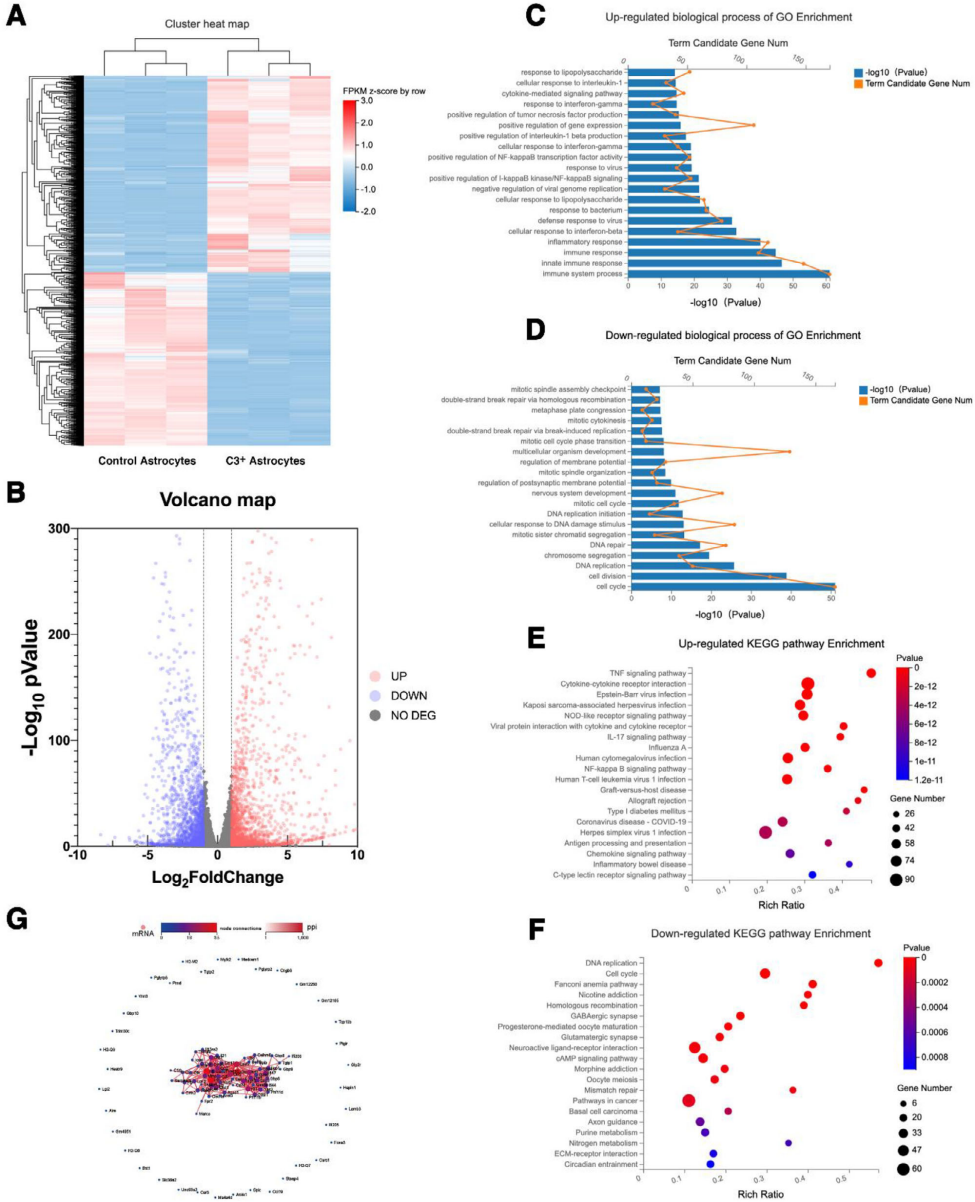
The expression of inflammatory, immune, and chemotactic signaling pathways is up‐regulated in C3^+^ astrocytes. A) Hierarchical clustering of mRNA sequencing showed mRNA differential expression between C3^+^ astrocytes and the control astrocyte group. B) The volcano map was performed for the alteration of differentially expressed mRNAs of C3^+^ astrocytes and the control astrocyte group. C) Up‐regulated biological process of GO Enrichment. D) Down‐regulated biological process of GO Enrichment. E) Up‐regulated KEGG pathway Enrichment. F) Down‐regulated KEGG pathway Enrichment. G) Performing Network Interaction Analysis on the Top 100 Differentially Expressed Genes.

To further explore the potential mechanisms by which C3^+^ astrocytes affect the proliferation, survival, and differentiation of NSCs, we analyzed the transcriptome differences between C3^+^ astrocytes and normal astrocytes. We found differential expression (|log2FC| ≥1, *p*‐value≤0.05) between C3^+^ astrocytes and normal astrocytes, with 1669 genes upregulated and 1471 genes downregulated (**Figure** **6**A,B). Gene Ontology (GO) biological process enrichment analysis showed that, compared to normal astrocytes, the upregulated genes in C3^+^ astrocytes were primarily enriched in biological process related to inflammatory response, immune response, and immune system response (Figure 6C), while downregulated genes were primarily enriched in DNA repair, cell cycle, and cell division (Figure 6D). KEGG analysis further revealed that the upregulated genes were primarily enriched in pathways such as the TNF signaling pathway, IL‐17 signaling pathway, chemokine signaling pathway, NOD‐like receptor signaling pathway, and NF‐kappaB signaling pathway (Figure 6E). In contrast, the downregulated genes were predominantly associated with DNA replication, cell cycle, and Mismatch repair (Figure 6F). Network analysis of the top 100 differentially expressed genes identified IL‐1b, IL‐6, Cxcl10, Cxcl9, Cxcl2, among others, as central nodes in the network, exhibiting a high degree of interactions (Figure 6G).

### 2.7 GLP1R Agonist Semaglutide Blocked the Astrocyte Conversion to the C3^+^ Inflammatory Phenotype by Inhibiting Microglia Activation

**Figure 7:**
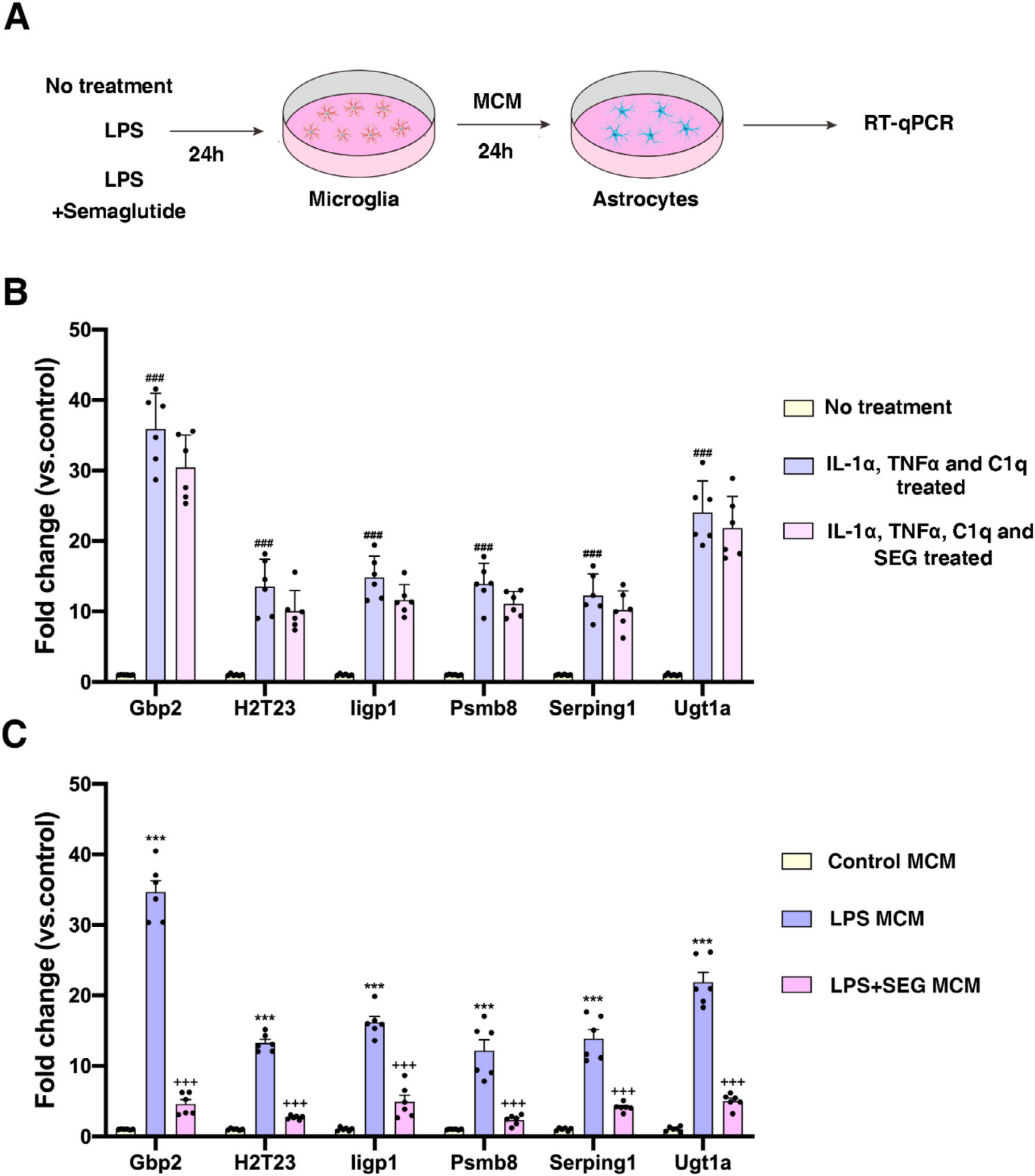
GLP1R agonist semaglutide blocked the conversion of astrocytes to the C3^+^ inflammatory phenotype by inhibiting the activation of microglia. A) The flowchart for Cell Experiment 2. B) Expression of C3‐related genes after Il‐1α + TNF‐α + C1q + SEG or Il‐1α + TNF‐α + C1q treatment. C) Expression of C3‐related genes after treatment with LPS MCM or LPS + SEG MCM. ^###^ *p*<0.001 compared with the No treatment group; ^***^ *p*<0.001 compared with the control MCM group; ^+++^ *p*<0.001 compared with the LPS MCM group. (*n* = 6 samples per condition; one‐way ANOVA with Tukey post‐test).

The flowchart for Cell Experiment 2 is shown in **Figure** **7**A. To investigate whether the GLP1R agonist semaglutide can directly block the phenotypic alterations in astrocytes, we treated astrocytes with IL‐1α+TNFα+C1q or IL‐1α+TNFα+C1q+semaglutide. RT‐qPCR results showed that the GLP1R agonist semaglutide did not inhibit the phenotypic changes of astrocytes induced by IL‐1α, TNFα, and C1q (Figure 7B). Given that C3^+^ astrocytes can be induced by classically activated neuroinflammatory microglia, we sought to explore whether semaglutide could exert its effects on astrocytes via microglia. Immunofluorescence staining was used to identify primary cultured microglia (Figure S3D, Supporting Information). LPS intervention induced a morphological transformation of microglia into an amoeboid shape (Figure S4A, Supporting Information). RT‐qPCR analysis revealed that, compared to normal microglia, LPS‐treated microglia exhibited increased expression of IL‐1α, TNFα, and C1q (Figure S4B, Supporting Information). Similarly, RT‐qPCR results demonstrated that LPS microglia conditioned medium (LPS MCM) increased the expression of neurotoxic astrocyte‐related genes (Serping1, Gbp2, Iigp1, H2‐T23, Ugt1a, Psmb8) in astrocytes (Figure S4C, Supporting Information), indicating that LPS MCM can induce the transformation of astrocytes into a C3^+^ phenotype.

Interestingly, compared to the LPS MCM group, the LPS+SEG MCM group reduced the expression of neurotoxic astrocyte‐related genes (Serping1, Gbp2, Iigp1, H2‐T23, Ugt1a, Psmb8) (Figure 7C). These findings suggested that semaglutide can block the phenotypic transformation of astrocytes by inhibiting microglial activation, rather than directly altering astrocyte phenotype.

## 3 Discussion

In this study, we found that the combined treatment of NSCs transplantation with SEG more effectively ameliorated motor dysfunction in PD mice compared to NSCs transplantation alone. This combination also reduced the expression of C3^+^ reactive astrocytes and promoted the survival, migration, and differentiation of the transplanted NSCs. In vitro studies suggested that SEG might exert its effects by inhibiting microglial activation, thereby preventing the transformation of astrocytes into the C3^+^ neurotoxic phenotype. Further investigations revealed that C3^+^ reactive astrocytes significantly affect the survival, proliferation, and differentiation of NSCs.

Treatment for PD includes medication, surgery, physical therapy, psychological counseling, and nursing care.^[^ ^2^, ^4^ ^]^ Despite extensive research, there is no conclusive evidence to support that current treatment methods slow the progression of the disease.^[^ ^1^, ^3^, ^5^ ^]^ Therefore, there is a critical need to explore novel therapeutic strategies for PD. Among emerging approaches, NSCs transplantation therapy shows promise as a potential treatment for PD. However, the clinical application of NSCs transplantation therapy faces significant challenges, particularly concerning the low survival rate of transplanted cell,^[^ ^29^, ^30^ ^]^ a problem likely associated with the microenvironment surrounding the transplanted cells.^[^ ^12^ ^]^

Astrocytes, the most abundant and widely distributed glial cells in the brain, form a crucial part of the microenvironment.^[^ ^23^, ^31^ ^]^ Autopsy studies have found elevated expression of C3^+^ astrocytes in the pathological regions of PD brain tissue.^[^ ^20^, ^23^ ^]^ In line with these findings, we observed a substantial presence of C3^+^ reactive astrocytes in the striatum of 6‐OHDA‐induced PD mouse models. Our observations indicated that newly generated cells in the striatum of PD mice gradually undergo cell death over time, suggesting that the activation of neural progenitor cells following pathological stimulation is transient. Clearly, the impairment of the nigrostriatal system is detrimental to the survival, proliferation, and directed differentiation of NSCs. Based on this, we hypothesized that C3^+^ astrocytes, as a crucial component of the central nervous system microenvironment, may affect the survival and integration of transplanted NSCs through non‐cell‐autonomous toxicity. Consequently, the development of drugs that inhibit the transformation of astrocytes to C3^+^ astrocytes might enhance the efficacy of NSCs transplantation therapy for PD.

GLP1R agonists protect against the loss of dopaminergic neurons and behavioral deficits in the α‐synuclein preformed fibril mouse model of PD by preventing microglial‐mediated conversion of astrocytes to a neurotoxic phenotype.^[^ ^27^ ^]^ SEG, a GLP1R agonist, entered Phase II clinical trials for PD patients in 2019 (NCT03659682), and has been approved by the U.S. Food and Drug Administration and the European Medicines Agency for the treatment of type 2 diabetes in adults.^[^ ^32^ ^]^ In summary, SEG is a safe and promising candidate for inhibiting the conversion of astrocytes to the C3^+^ neurotoxic phenotype.

We further investigated whether the combination of NSC transplantation with SEG could improve the therapeutic effects of NSCs transplantation in PD. Our results indicated that the combination treatment of SEG and NSCs transplantation can further improve motor dysfunction in PD mice compared to NSC transplantation alone. Previous studies have suggested that SEG may exert various effects, such as alleviating inflammation, reducing lipid peroxidation, decreasing α‐synuclein accumulation, inhibiting apoptotic pathways, and increasing the expression of autophagy‐related proteins.^[^ ^33^, ^34^, ^35^, ^36^ ^]^ Research has shown that GLP1R agonists can alleviate motor dysfunction in PD animal models.^[^ ^27^, ^37^, ^38^ ^]^ Several clinical studies have confirmed that GLP1R agonists can improve motor function in PD patients.^[^ ^39^, ^40^, ^41^ ^]^ The safety profile and multifunctionality of SEG make it a promising candidate for future PD treatment. However, the specific mechanisms by which GLP1R agonists enhance the effectiveness of NSCs transplantation therapy in PD remain poorly understood. In vivo imaging techniques to track DiD fluorescent dye‐labeled NSCs, combined with fluorescence imaging experiments, revealed that SEG combined with NSCs transplantation therapy increased the survival, migration, and differentiation of transplanted cells. Further studies showed that SEG combined with NSCs transplantation therapy reduced the astrocyte activation in the brains of PD mice, thereby preventing the transformation of astrocytes into the toxic C3^+^ phenotype. Based on these findings, we hypothesized that SEG might play a role by regulating the phenotypic transition of astrocytes. To investigate how the GLP1R agonist SEG regulates the phenotypic conversion of astrocytes, we conducted a series of in vitro experiments. The results indicated that SEG blocks the conversion of astrocytes to the C3^+^ phenotype by inhibiting microglial activation.

Our animal studies have shown a significant presence of C3‐reactive astrocytes in PD mice, and these C3‐reactive astrocytes may influence the survival and differentiation of NSCs. To further confirm the impact of C3‐reactive astrocytes on NSCs, we conducted conditional co‐culture experiments in vitro. The results of these in vitro experiments indicated that the conditioned medium from C3‐reactive astrocytes increased NSC death, inhibited their proliferation, and reduced their differentiation into neurons/dopaminergic neurons. Our research suggests that C3‐reactive astrocytes lose their normal nutritional support function and instead exert toxicity on NSCs, impairing their survival, proliferation, and differentiation into mature cells.

To understand the potential mechanisms by which C3^+^ reactive astrocytes affect NSCs, we performed transcriptomic analysis comparing normal astrocytes and C3^+^ reactive astrocytes. Our data indicated that various biological processes, such as inflammatory and immune responses, are increased in C3^+^ reactive astrocytes. Pathways including the TNF signaling pathway, IL‐17 signaling pathway, chemokine signaling pathway, NOD‐like receptor signaling pathway, and NF‐kappaB signaling pathway were found to be enriched in C3^+^ reactive astrocytes. Inflammatory cytokines (e.g., TNF and IL‐6) and chemokines (e.g., Cxcl10, Cxcl9, and Cxcl2) were increased and centrally positioned in network interaction analysis, showing a high degree of interactivity. Growing evidence suggests that excessive activation of inflammatory and immune responses may have negative effects on the survival, proliferation, and differentiation of NSCs,^[^ ^42^, ^43^, ^44^, ^45^, ^46^ ^]^ especially pathways like NF‐kappaB signaling and IL‐17 signaling. C3^+^ reactive astrocytes may affect NSCs through excessive inflammatory responses.

A recent study identified that the TNF‐NF‐κB‐p53 axis restricts in vivo survival of hPSC‐derived dopamine neurons in PD models.^[^ ^47^ ^]^ While our findings highlighted that the enrichment of TNF‐NF‐κB signaling in host C3+ astrocytes may affect transplanted NSCs' survival through non‐cellular autonomous toxicity. These parallel mechanisms may interact to form a detrimental feedback loop that further compromises graft outcomes.

## 4 Conclusion

The transplantation of NSCs combined with SEG can improve motor dysfunction in PD mice, reduce the conversion of astrocytes to the C3^+^ phenotype, and enhance the survival, migration, and differentiation of transplanted NSCs. Overall, the combination of NSCs transplantation with SEG demonstrated greater efficacy in treating PD compared to NSCs transplantation alone. In vitro studies suggested that the GLP1R agonist SEG may inhibit the activation of microglial cells, thereby preventing the transformation of astrocytes into the C3^+^ neurotoxic phenotype. C3^+^ astrocytes lose their normal supportive functions and exert toxic effects on NSCs, severely impairing their survival, proliferation, and differentiation capabilities.

## 5 Experimental Section

### Animals and Experimental Design

Adult male C57BL/6J mice (8‐10‐week‐old) purchased from the Experimental Animal Center of Chong Qing Medical University (Chongqing, China) were used in vivo studies. All animal procedures complied with the Guide for the Care and Use of Laboratory Animals published by the US National Institutes of Health (NIH), and approved by the Institutional Animal Care and Use Committee at Chong Qing Medical University (License Number: SYXK 2022‐0010). Mice were housed in a standardized environment with a humidity of 60–65% and controlled temperature at 23 ± 2 °C with a 12 h light‐dark cycle.

### Animals and Experimental Design—*Animal Experiment 1*

Mice were randomly divided into two groups: the control group (Control group) and the PD model group (PD group). The experimental procedure for this part of the study is as follows: both groups of mice were intraperitoneally injected with EDU (50 mg/kg, Beyotime, China, Cat#ST067) on days 1–3 after surgery. On day 28 after surgery, the mice underwent an apomorphine (APO)‐induced rotation test, EDU staining, immunofluorescence staining, and western blot; on day 42, EDU staining and immunofluorescence staining were performed.

### Animals and Experimental Design—*Animal Experiment 2*

Mice were randomly divided into 5 groups: the control group (Control group), PD model group (PD group), PD model treated with GLP1R agonist semaglutide by intraperitoneal injection group (SEG group), PD model with NSCs transplantation group (NSCs group), and PD model with SEG combined with NSCs transplantation group (SEG+NSCs group). The experimental procedure for this part of the study is as follows: mice in each group underwent NSCs transplantation or injection of an equal amount of solvent on day 28 after surgery. The SEG group and SEG+NSCs group received intraperitoneal injection of semaglitide (MCE, USA, HY‐114118) 2 h before transplantation, once every 2 days for 4 consecutive weeks. The NSCs group and SEG+NSCs group underwent in vivo imaging experiments on day 56. Behavioral experiments were conducted from day 56 to day 70. Immunofluorescence staining and Western Blot were performed after the completion of behavioral experiments.

### Construction of PD Mouse Model

Mice were intraperitoneally injected with APO (0.5 mg kg^−1^, Sigma‐Aldrich, Cat#PHR2621), and those without rotational behavior were randomly assigned to either the control group or the PD group. Thirty minutes prior to surgery, desipramine (Sigma‐Aldrich, Cat#D3900) and pargyline (Sigma‐Aldrich, Cat#P8013) were administered intraperitoneally to the mice. Anesthesia was induced using sodium pentobarbital (40 mg kg^−1^, ip). Following anesthesia, the fur on the scalp was shaved, and the mice were secured in a stereotaxic apparatus. The scalp was disinfected with povidone‐iodine, then incised along the midline to expose the skull. Connective tissue on the skull surface was carefully removed using hydrogen peroxide to expose the bregma. 6‐OHDA (10 µg µL^−1^, Sigma‐Aldrich, Cat#H4381) was injected into the striatum at the following coordinates: AP: +0.5 mm, ML: −2.0 mm; DV: −2.0, and −3.0 mm. At each depth, 0.4 µL of solution was injected at a rate of 0.1 µL min^−1^. In the control group, an equal volume of vehicle (ascorbate‐saline solution) was injected using the same procedure. After injection, the needle was left in place for 5 minutes before being slowly withdrawn. The scalp was disinfected and sutured, and mice were placed on a heated pad until recovery from anesthesia. Four weeks post‐surgery, APO (0.5 mg kg^−1^) was injected intraperitoneally to induce rotational behavior. Rotational behavior was observed and recorded for 10 min following APO administration. An average rotational speed ≥ 7 r min^−1^ was considered indicative of successful PD model establishment.

### Immunofluorescence Staining

Brain slices or cell slides were fixed with 4% PFA for 15 min and incubated in 0.3% Triton X‐100 solution at 37 °C for 30 min. They were then incubated with goat serum (Boster, China, Cat#AR0009) or donkey serum at room temperature for 2 h. Brain slices were incubated overnight at 4 °C with antibodies against TH (Proteintech, China, Cat#25859‐1‐AP), GFAP (Proteintech, China, Cat#16825‐1‐AP), C3d (R&D Systems, Cat#AF2655), NeuN (Abcam, UK, Cat#ab177487), Nestin (Abcam, UK, Cat#ab6142), TuJ1 (Abcam, UK, Cat#ab78078), and Iba1 (Abcam, UK, Cat# ab178846). After washing three times in PBS, the brain slices or cell slides were incubated with fluorescent‐conjugated secondary antibodies at room temperature for 2 h. Fluorescent images were acquired using a confocal scanning system (ZEISS, Germany).

### Western Blot

Dissect the striatum from brain tissue and lyse in RIPA lysis buffer (Beyotime, China, CAT#P0013B) supplemented with protease inhibitors (PMSF, Beyotime, China, CAT#ST506) and phosphatase inhibitor cocktail A (Beyotime, China, CAT#P1082) on ice. The protein concentration of each sample was determined using a BCA assay kit (Beyotime, China, CAT#P0012).

Electrophoresis was conducted by a 10% SDS‐polyacrylamide gel, and transferred protein samples were transferred to PVDF membranes (Millipore, Cat# IPVH00010). The following primary antibodies were used for incubation overnight at 4 °C: rabbit anti‐beta‐actin (Proteintech, China Cat# 81115‐1‐RR), rabbit anti‐tyrosine hydroxylase (TH) (Proteintech, China, Cat#25859‐1‐AP), rabbit anti‐GFAP (Proteintech, China, Cat#16825‐1‐AP), and goat anti‐C3d (R&D Systems, Cat#AF2655). All membranes were incubated with HRP‐conjugated Affinipure Rabbit Anti‐Goat IgG (H+L) (Proteintech, China, Cat#RGAM001) or HRP‐conjugated Affinipure Goat Anti‐Rabbit IgG (H+L) (Proteintech, China, Cat#SA00001‐2) for 1 h. The immunoblots were detected using an enhanced chemiluminescence (ECL) kit (Advansta, USA Cat#K‐12045‐D10) and analyzed with Image J.

### EDU Staining

Brain slices or cell slides were fixed with 4% PFA for 15 min and incubated in 0.3% Triton X‐100 solution at 37 °C for 30 min. Then the Click reaction mixture was added and incubated at room temperature, protected from light, for 30 min. The Click reaction mixture (Beyotime, China, Cat#C0078) was prepared in the following component order and volume ratio: Click Reaction Buffer 860 µL, CuSO4 40 µL, Azide 594 2 µL, Click Additive Solution 100 µL. After washing three times in PBS, the brain slices or cell slides were incubated with DAPI (Beyotime, China, CAT#C1005) at room temperature for 10 min. Images were acquired using a confocal scanning system (ZEISS, Germany).

### NSCs Transplantation

The PD mice were anesthetized and secured on a stereotaxic apparatus. The NSCs suspension (2 µL, 2 × 10^5^ cells, labeled with DiD (MCE, USA, HY‐D1028), 0.4 µL min^−1^) or PBS were stereotactically injected into the right striatum of the mice using the following coordinates relative to the bregma: anterior‐posterior (AP) −0.5 mm, mediolateral (ML) +2.0 mm, and dorsoventral (DV) −3.0 mm from the dura. After the injection, the needle was left in place for 5 min, then slowly withdrawn. The skin was disinfected, sutured, disinfected again, and the mice were placed on a warming pad until they recovered consciousness.

### In Vivo Imaging

The mice were placed in an anesthesia chamber and anesthetized with inhaled isoflurane. Once anesthetized, they were removed and placed on the imaging stage of the in vivo imaging system. The appropriate imaging mode and parameters were selected based on the experimental requirements. The imaging was performed following the instrument's instructions, with attention paid to the mice's positioning and surface luminescence to ensure imaging accuracy and stability. IndiGo software was used to process and analyze the images and data obtained from the imaging.

### Behavioral Test—*Pole Climbing Test*

The mice were placed in an anesthesia chamber and anesthetized with inhaled isoflurane. Once anesthetized, they were removed and placed on the imaging stage of the in vivo imaging system. Selected the appropriate imaging mode and parameters based on the experimental requirements. Follow the instrument's instructions to perform imaging, paying attention to the mice's positioning and surface luminescence to ensure imaging accuracy and stability. Use the IndiGo software to process and analyze the images and data obtained from the imaging.

### Behavioral Test—*Rotarod Test*

Training Period: The mice were placed on the rod of the rotarod apparatus to acclimate to the environment for 5 min. Then, the initial rod speed was set to 4 rpm, acceleration to 0.1 rpm s^−1^, maximum speed to 30 rpm, and single training time to 5 min. The mice were trained 3 times a day for 2 consecutive days to acclimate them to the environment and improve the reliability and accuracy of the test.

Testing Period: The mice were placed on the rod of the rotarod apparatus to maintain balance. A total of 5 min was set as the test endpoint. Press the start button and record the latency to fall and the speed at the time of fall for each mouse. Each mouse was tested 3 times, with a 30‐min interval between tests. The average latency to fall and the speed at the time of fall from the three tests were used as the experimental results.

### Behavioral Test—*Grip Strength Test*

The middle part of the mouse's tail was grasped, and the mouse was placed on the grid, allowing its front and hind paws to touch the grid before measurement, ensuring that the trunk and the grid were level. The tail of the mouse was gently pulled from the top of the grid, and the grip force was recorded. This process was repeated 3 times, recording the grip force measurements for all limbs (maximum grip force and average grip force). The average of the three tests was used as the experimental result.

### Behavioral Test—*Balance Beam Test*

The day before the formal test, the mice were trained on the balance beam three times. During the formal test, the time taken for the mouse to walk from one end of the balance beam to the other was recorded. Each mouse needs to undergo the experiment 3 times to reduce individual differences and errors. There was a 30‐min interval between experiments to avoid the influence of animal fatigue on the experimental results.

### Behavioral Test—*Gait Test*

The coordination of the natural movement behavior of mice was evaluated using the gait experiment. The gait analysis system (CatWalk) consisted of a transparent runway and an underlying camera to record the footprints of mice. One day before the formal experiment, mice were allowed to freely walk or run on the runway to acclimate to the equipment. During the experiment, mice freely passed through the set‐length detection channel. The footprint refraction technique of the internal light source was utilized to process the footprints in the videos captured by the camera, evaluating the movement under natural walking conditions of mice. The entire process was conducted in a darkroom environment, with each mouse undergoing the experiment three times. The collected data was ensured to be clear, accurate, and continuous. Statistical analysis was performed on the time taken to pass a specific distance (Duration), the number of steps per unit time (Cadence), average speed (Average Speed), maximum rate of change (Maximum Variation), support phase time (Stand), stride length (Stride Length), and step cycle (Step Cycle).

### Primary Cell Culture and Experimental Design

According to previous studies, NSCs were extracted from 0–2‐day‐old neonatal C57BL/6J mice.^[^ ^48^ ^]^ Brains were harvested from neonatal (P0–P2) C57BL/6J mice and immediately placed in pre‐chilled D‐Hank's solution to rinse off blood and debris. The brains were then transferred to cold DMEM/F12 medium, and the meninges and blood vessels were carefully removed. The midbrain region was dissected and transferred to another dish containing pre‐chilled D‐Hank's solution for further rinsing. The tissue was then minced and digested in 0.125% trypsin at 37 °C for 15 min. Digestion was terminated with DMEM/F12 containing 10% fetal bovine serum (FBS). The resulting suspension was filtered through a cell strainer to remove undigested tissue fragments. The filtered cell suspension was centrifuged at 1000 rpm for 5 min, and the supernatant was discarded. The cell pellet was gently resuspended in NSC culture medium (DMEM/F12 supplemented with 2% B27, 20 ng mL^−1^ epidermal growth factor (EGF), and 20 ng mL^−1^ basic fibroblast growth factor (bFGF)). The cell density was adjusted to 5 × 10^5^ cells mL^−1^ using a cell counter. Cells were seeded into T25 or T75 flasks and cultured at 37 °C in a 5% CO_2_ incubator.

To determine the effects of C3^+^ astrocytes on the survival, proliferation, and differentiation of NSCs, control astrocyte conditioned medium (Control ACM) and C3^+^ astrocyte conditioned medium (C3 ACM) are collected and used for the conditional co‐culture of NSCs. Subsequently, Calcein/PI cell viability and cytotoxicity assays, EDU staining, immunofluorescence staining, and transcriptomic analysis were performed.

To determine whether the SEG directly blocks astrocyte phenotype changes, astrocytes were treated with IL‐1α+TNFα+C1q or IL‐1α+TNFα+C1q+semaglutide (30 nm) for 24 h, followed by RT‐qPCR to detect the expression of neurotoxic astrocyte‐related genes (Serping1, Gbp2, Iigp1, H2‐T23, Ugt1a, Psmb8). To determine whether SEG blocks astrocyte phenotype changes through the action of microglia, conditioned media from microglia stimulated with Lipopolysaccharide (LPS) or LPS+semaglutide (30 nm) (LPS MCM or LPS+SEG MCM) were used to treat astrocytes for 24 h, followed by RT‐qPCR to detect the expression of neurotoxic astrocyte related genes (Serping1, Gbp2, Iigp1, H2‐T23, Ugt1a, Psmb8).

### Real‐Time Quantitative PCR

Total RNA was extracted using the Cell Total RNA Isolation Kit (FOREGENE, China, RE‐03113), and cDNA was synthesized from total RNA by reverse transcription using the RT Master Mix for qPCR II (MCE, USA, HY‐K0511A). RT‐qPCR was processed with SYBR Master Mix (MCE, USA, HY‐K0523) by using a CFX Real‐Time PCR Detection System (Bio‐Rad, CA). Primers were listed in Table S1 (Supporting Information). The expression level of the target gene is calculated relative to β‐actin (2^‐ΔΔCT).

### Calcein AM/PI Staining

The culture medium was removed from the live cells. An appropriate volume of Calcein AM/PI detection solution (prepared as follows: 1 µL Calcein AM (1000X), 1 µL PI (1000X), 1 ml detection buffer, prepared and used immediately) was added, and the cells were incubated at 37 °C in the dark for 30 min. Immediately observed the staining effect under a fluorescence microscope and took pictures.

### RNA Sequencing and Differentially Expressed Gene Analysis

Total RNA was extracted from cells, and then Nano Drop and Agilent 2100 Bioanalyzer (Thermo Fisher Scientific, MA, USA) were used for the identification and quantification of total RNA. RNA library construction and subsequent RNA sequencing were completed by BGI Group in Shenzhen, China. Sequencing data was filtered using SOAPnuke, and the original sequencing data was mapped to the reference genome using HISAT2. The original data were compared with the database established by BGI Group, and gene expression levels were calculated using RSEM (v1.3.1). Based on the gene expression differences in different samples, a heatmap was drawn using phatmap (v1.0.8). Differential expression analysis was performed using DESeq2 (v1.4.5). To further understand the phenotypic changes, enrichment analysis of annotated differentially expressed genes is conducted using GO (http://www.geneontology.org/) and KEGG (https://www.kegg.jp/) based on the Hypergeometric test. All analyses were performed on the Dr. Tom analysis system built by BGI Group in Shenzhen, China.

### Statistical Analysis

All data were analyzed using GraphPad Prism 8.0 (GraphPad, USA) software. The normality and homogeneity of variance of the data are analyzed using the Kolmogorov‐Smirnov test and the Shapiro‐Wilk test. Comparisons between the two groups were performed using Student's t‐test. Comparisons among multiple groups were performed using one‐way analysis of variance (One‐way ANOVA) to assess statistical significance between groups, followed by the Tukey post‐hoc test for further analysis. All quantitative data were presented as mean ± standard error of the mean (mean ± SEM). A *p*‐value of <0.05 was considered statistically significant (*p* < 0.05).

## Conflict of Interest

The authors declare no conflict of interest.

## Supporting information



Supporting Information

## Data Availability

The data that support the findings of this study are available from the corresponding author upon reasonable request.
