# Supplementary material for: The GLP1R Agonist Semaglutide Inhibits Reactive Astrocytes and Enhances the Efficacy of Neural Stem Cell Transplantation Therapy in Parkinson's Disease Mice
Source: Adv Sci (Weinh). 2025 Aug 28;12(43):e17664. doi: 10.1002/advs.202417664 (PMC12631936; doi:10.1002/advs.202417664)
Supplement: Supplementary file 1 — Supporting Information [file ADVS-12-e17664-s001.docx]

Supporting Information

Title: The GLP1R Agonist Semaglutide Inhibits Reactive Astrocytes and Enhances the Efficacy of Neural Stem Cell Transplantation Therapy in Parkinson's Disease Mice

Dan Song, Xiaoya Zou, Di Ma, Yuying Zhao, Tingting Liu, Bibiao Shen, Oumei Cheng*

**
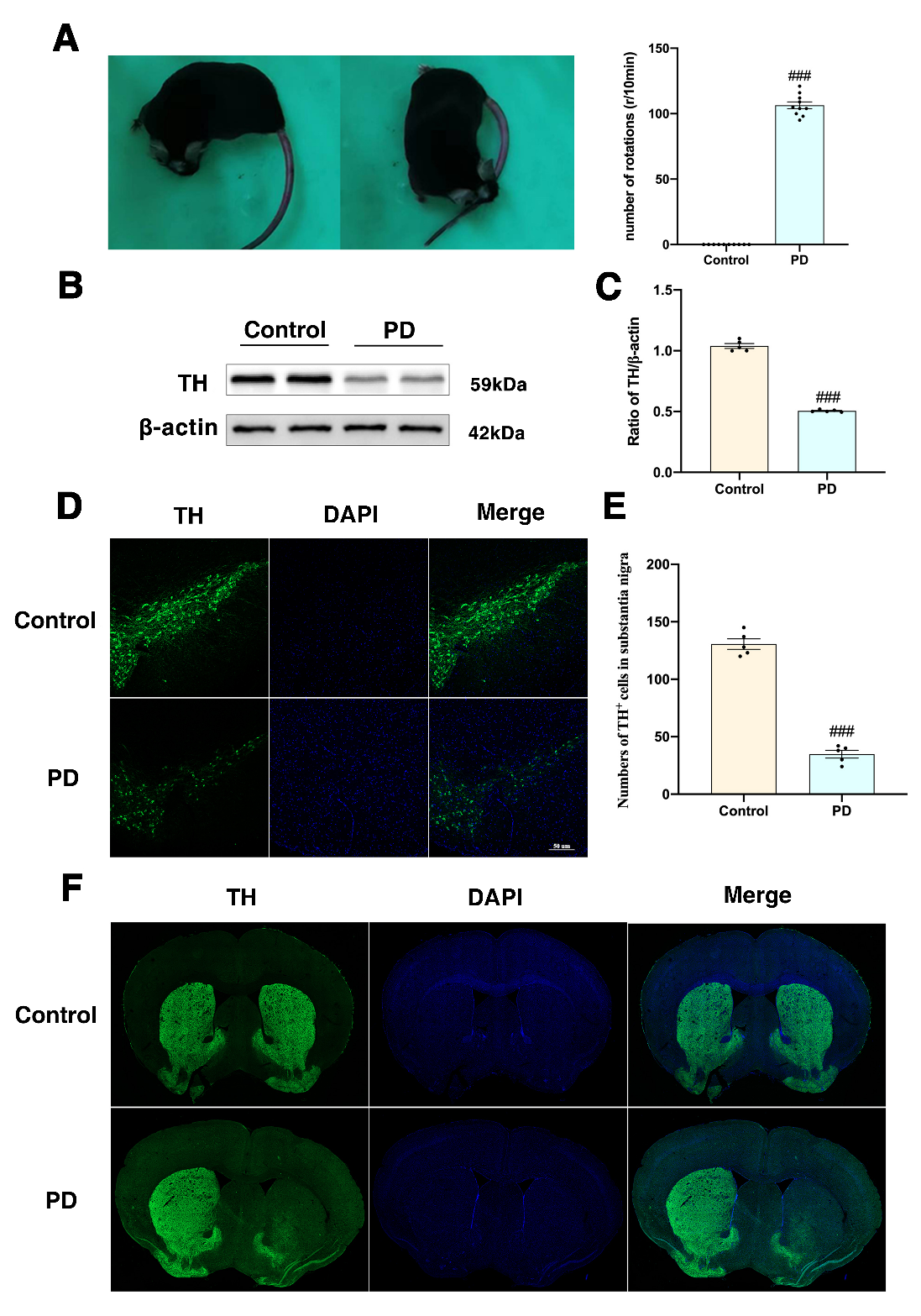
**

**Figure S1:** 6-OHDA induced PD mouse model. (A) APO induced mouse rotation experiment and statistics of rotation times. (B) The representative bands of TH expression in striatum of each group were detected by Western blot. (C) Statistical graph of TH expression in striatum detected by Western Blot. (D) Representative immunofluorescence image of TH^+^ cells expression in mouse substantia Nigra. Scale bar: 50 μm. (E) Quantitative analysis statistical graph indicates a significant decrease in the number of TH^+^ cells in the PD group of mice. (F) Brain slice scanning reveals a significant decrease in TH expression in the striatum of mice in the PD group. ###p<0.001 compared with the Control group. (n=10/5 samples per condition; unpaired Student’s t-test with Kolmogorov-Smirnov test was used to compare the two groups).


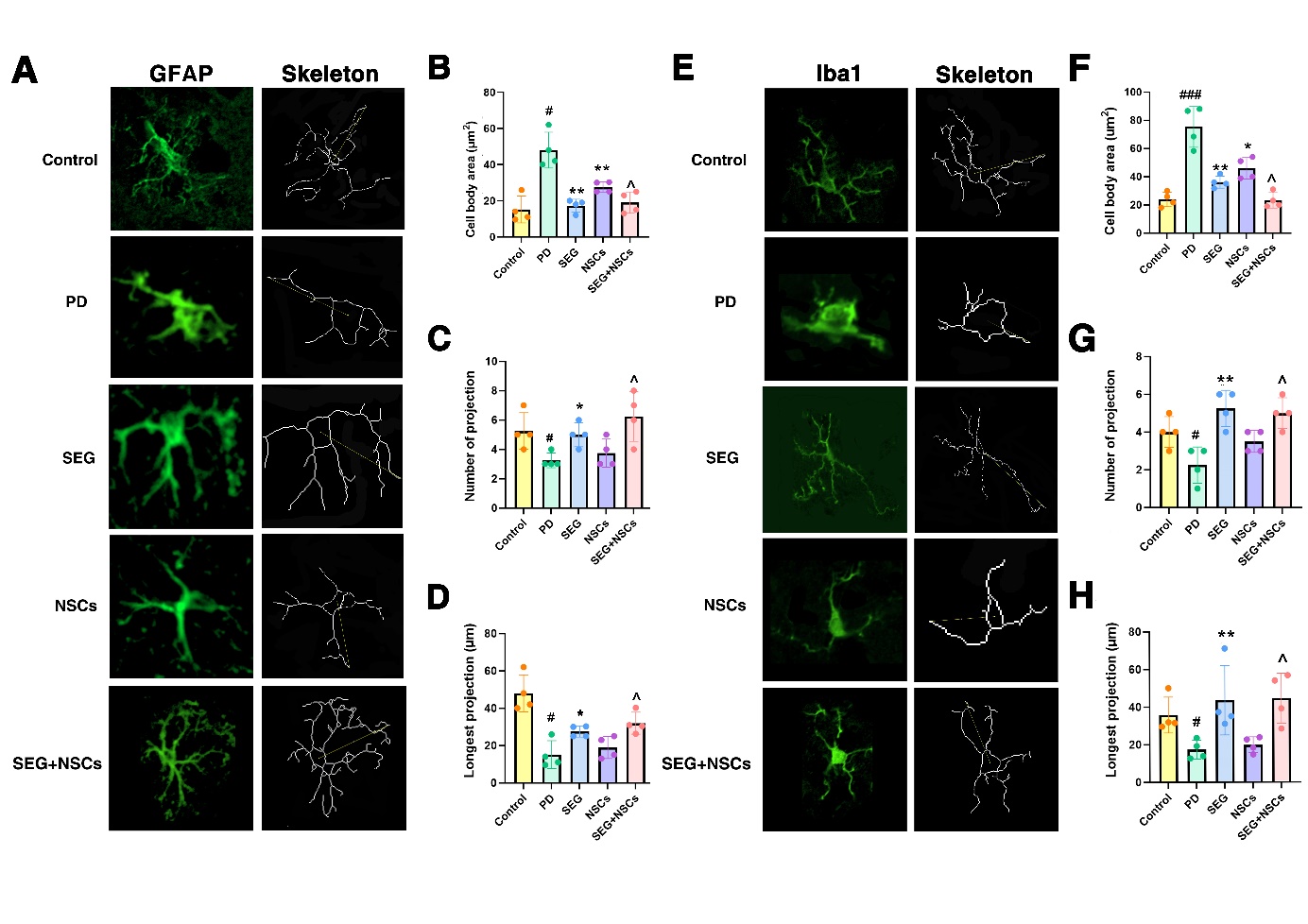


**Figure S2:**The effect of GLP1R agonist semaglutide on the morphology of astrocytes and microglia. (A) The morphological changes of astrocytes. (B) The cell body area of astrocytes. (C) The number of projections of astrocytes. (D) The longest projections of astrocytes. (E) The morphological changes of microglia. (F) The cell body area of astrocytes. (G) The number of projections of microglia. (H) The longest projections of microglia. #p<0.05 compared with the Control group; ###p<0.001 compared with the Control group;*p<0.05 compared with the PD group; **p<0.01 compared with the PD group; ^p<0.05 compared with the NSCs group. (n=4 samples per condition; one-way ANOVA with Tukey post-test).


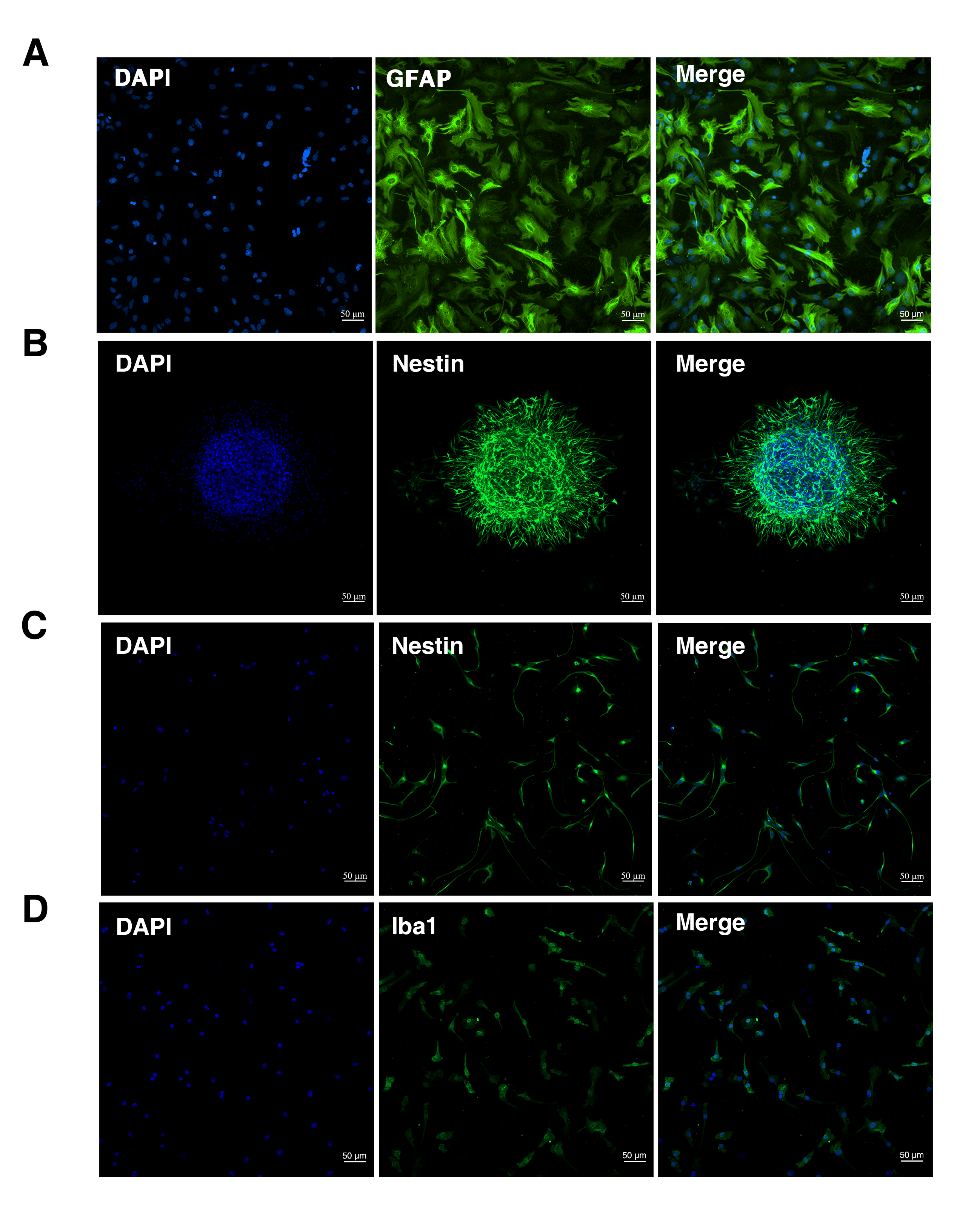


**Figure S3:** Identification of primary cultured cells. (A) The astrocytes showed typical GFAP immune reactivity. Scale bar: 50 μm. (B) Neurospheres have Nestin immunoreactivity typical of NSCs. Scale bar: 50 μm. (C) The individual cell exhibits Nestin immunoreactivity typical of NSCs. Scale bar: 50 μm. (D) The microglial cells showed typical Iba1immune reactivity. Scale bar: 50 μm.


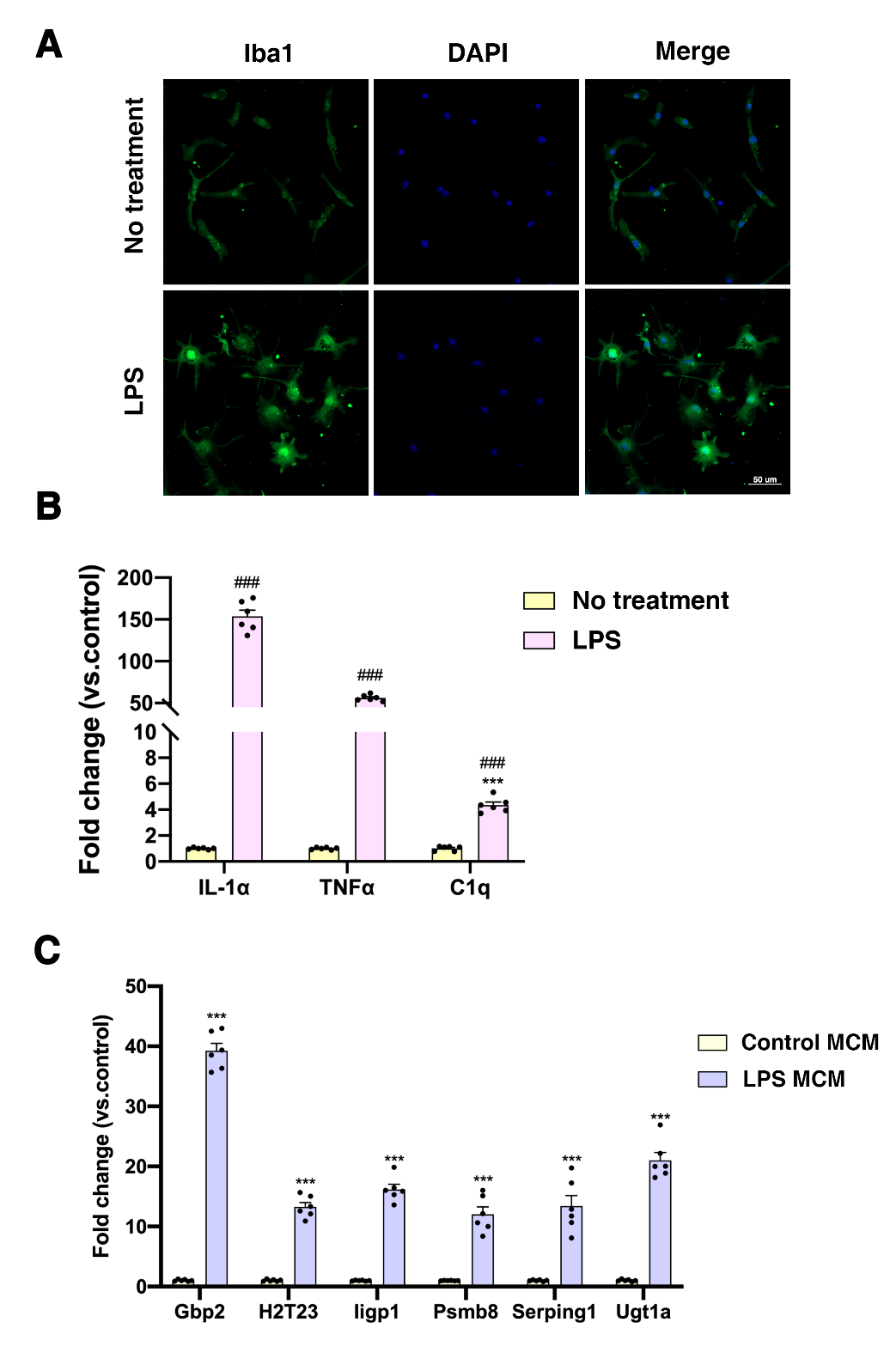


Figure S4: C3^+^ reactive astrocytes can be induced by classically activated neuroinflammatory microglia. (A) LPS activation alters the morphology of microglia. Scale bar: 50 μm. (B) LPS -activated microglia increased the expression of IL-1α, TNF-α and C1q genes. (C) LPS MCM treatment increased the expression of C3-related. ###p<0.001 compared with the No treatment group; ***p<0.001 compared with the control MCM group. (n=6 samples per condition; unpaired Student’s t-test with Kolmogorov-Smirnov test was used to compare the two groups).

Table S1: RT-qPCR Primers

| **Gene name** | **Forward primer** | **Reverse primer** |
| --- | --- | --- |
| **β-actin** | TATGCTCTCCCTCACGCCATCC | GTCACGCACGATTTCCCTCTCAG |
| **Serping1** | ACAGCCCCCTCTGAATTCTT | GGATGCTCTCCAAGTTGCTC |
| **Gbp2** | GGGGTCACTGTCTGACCACT | GGGAAACCTGGGATGAGATT |
| **Iigp1** | GGGGCAATAGCTCATTGGTA | ACCTCGAAGACATCCCCTTT |
| **H2-T23** | GGACCGCGAATGACATAGC | GCACCTCAGGGTGACTTCAT |
| **Ugt1a** | CCTATGGGTCACTTGCCACT | AAAACCATGTTGGGCATGAT |
| **Psmb8** | CAGTCCTGAAGAGGCCTACG | CACTTTCACCCAACCGTCTT |
| **IL-1α** | AGATTCTGAAGAAGAGACGGCTGAG | GGTAGGTGTAAGGTGCTGATCTGG |
| **TNF-α** | ACGCTCTTCTGTCTACTGAACTTCG | TGGTTTGTGAGTGTGAGGGTCTG |
| **C1q** | CCTGGCAAACCTGGCAATGTG | TTTCACGCCCTTCAGTCCTTGG |
